# Efficacy of a prepared tissue culture-adapted vaccine against *Chlamydia psittaci* experimentally in mice

**DOI:** 10.14202/vetworld.2020.2546-2554

**Published:** 2020-11-28

**Authors:** J. El-Jakee, Mahmoud D. El-Hariri, Mona A. El-Shabrawy, Afaf A. Khedr, Riham H. Hedia, Eman A. Khairy, E. S. Gaber, Eman Ragab

**Affiliations:** 1 Department of Microbiology, Faculty of Veterinary Medicine, Cairo University, Egypt; 2 Department of Microbiology and Immunology, National Research Center, El Dokki, Cairo, Egypt; 3 Central Laboratory for Evaluation of Veterinary Biologics (CLEVB), Abbasia, Cairo, Egypt

**Keywords:** *Chlamydia psittaci*, human, immunofluorescence, inactivated vaccine, poultry, Vero cell

## Abstract

**Background and Aim:**

*Chlamydia psittaci* is an intracellular pathogen with a broad range of hosts and endemic in nearly all bird species as well as many mammalian species. Outbreaks contribute to economic losses, especially due to infection of pet birds, poultry, and livestock. Worse, the organism has a zoonotic effect, and transmission to humans results in severe illness. Therefore, proper control measures need to be applied. We conducted a trial for the preparation and evaluation of inactivated vaccine against *C. psittaci*.

**Materials and Methods:**

Three *C. psittaci* strains (accession nos.: KP942827, KP942828, and KP942829) were grown in embryonated chicken eggs and then propagated for purification in Vero cells. The immunization experiment was experimentally performed in mice, which then were challenged with a virulent *C. psittaci* strain.

**Results:**

The immunization trial revealed nearly 100% protection after the challenge. The histopathological and immunofluorescence examinations of internal organs revealed that the prepared killed vaccines can effectively reduce chlamydial infection and shedding in animals with the proper level of protection.

**Conclusion:**

Our vaccine can be used to control economic and financial losses resulting from avian chlamydiosis, especially those in poultry industries. The zoonotic transmission risk highlights the need for proper control measures.

## Introduction

*Chlamydia psittaci* is an intracellular bacterium that causes avian chlamydiosis disease. It is found in a wide range of birds, including companion, domestic, and wild birds. Cattle egret and hoopoe may be a reservoir for *Chlamydiaceae* species and thus shed the organisms in their excreta [1]. The prevalence of *C. psittaci* in lovebirds was investigated where the *omp*A gene of *C. psittaci* had been detected in fresh fecal droppings and conjunctival swabs [2]. Due to zoonotic transmission, the organism poses a significant public health risk. It was mentioned that at-risk people include bird breeders, pregnant women, workers at quarantine stations, poultry farmers, abattoir workers, veterinarians, pet shop workers, and employees and visitors of zoos, wildlife parks, petting zoos, and circuses [3]. A recent study stated that *C. psittaci* is reported to be the main etiologic agent in 1% of community-acquired pneumonia globally [4]. *Chlamydia* does not show strict host specificity. They infect more than 150 avian species, many mammalian species, and an increasing number of isolates from invertebrates. The avian strains were classified into six serovars (A-F) by means of serovar-specific monoclonal antibodies (mAbs) [5,6], while the mammalian strains were divided into nine immunotypes by means of an indirect immunofluorescence test [7].

In Egypt, there have been limited studies about the prevalence of *C. psittaci* in domestic birds, such as pigeons, turkeys, ducks, and chickens [8,9]. This enormous range of host species was matched by the diversity of recognized clinical conditions associated with chlamydial infections. Despite the ability of *C. psittaci* to induce a variety of disease syndromes, one clinical condition usually predominates in an outbreak or series of related clinical cases. The presenting condition and its severity depend on the strain and virulence of the agent, age, sex, physiological state, and host species, route of infection, degree of exposure to *Chlamydia* species, environment, and management factors [10]. *C. psittaci* is nearly endemic in the commercial poultry industry. Devastating outbreaks with high mortality occasionally occur, but respiratory signs without mortality characterize most outbreaks. Nevertheless, *C. psittaci* infections cause significant economic losses, and they are a threat to public health, particularly when this zoonotic agent infects e.g. poultry workers [11].

Reduction of avian Chlamydiosis will affect not only the disease and associated mortality but also it will also reduce the incidence of the carrier state as well as improve breeding performance in birds and mammals. The design of a vaccine and its successful implementation also have the potential to improve human health by decreasing the zoonotic risk and the risk of emergence of antibiotic-resistant strains [12,13].

Vaccination is the best approach for controlling the spread of chlamydial infections in animal and human populations. The main goal for vaccine research is to develop effective vaccines that induce sterile, lifelong, and heterotypic defensive immune responses. To date, the greatest success has been in developing whole organism-based killed or live-attenuated vaccines against the animal pathogens *Chlamydia* abortus and *Chlamydia felis* [14,15]. There have been approximately 23 vaccination trials (10.5%) against *C. psittaci* within a variety of different hosts, including mice, sheep, birds, Guinea pigs, and cats [16]. Inactivated or killed vaccines are more effective in reducing chlamydial shedding from birds with a good level of protective antibody titer, which creates an effective barrier for protection. Moreover, inactivated vaccines overcome the disadvantages of other types of *C. psittaci* vaccines because it is considered cheap and applicable [12]. DNA vaccination involves integrating the virus’ DNA into the DNA of the host cell chromosome to stimulate the generation of antibodies to the DNA, with the aim to induce an immune response that might cross-react with the host DNA [17].

The development of effective vaccines against chlamydial infections requires a clear understanding of the bacterial components that are essential to protective immunity; a suitable vaccine must be developed with these immunogenic elements along with effective delivery systems [18]. Hence, there is an urgent need for an efficient vaccination strategy against *C. psittaci*. In the present study, for the first time in Egypt, we tried to prepare an inactivated tissue culture vaccine against *C. psittaci*. Vero cells were used to propagate and purify the embryonated chicken egg (ECE) material for the growth of *C. psittaci* strains, which have been used as a bacterin to vaccinate mice as a model.

## Materials and Methods

### Ethical approval

Euthanasia steps and animals’ handling followed the Ethical Guidelines of the Cairo University Institutional Animal Care and Use Committee, under the number of CU/11/S/28/17.

### Study period and location

The research work and the immunization trial were carried out from April 2017 to May 2018 at Microbiology Department, Faculty of Veterinary medicine, Cairo University- Egypt.

### Microorganisms and seeds preparation

Three standard Egyptian birds’ strains of *C. psittaci* were used for vaccine preparation (accession nos.; KP942827, KP942828, and KP942829, which were kindly obtained from Dr. Eman Ragab, Faculty of Veterinary Medicine, Cairo University). Due to biological hazards regulations, there was difficulty in shipping any standard strains of *C. psittaci*, as it is categorized as a biological weapon. The strains used were ECE grown *C. psittaci* strains, which were inoculated into specific pathogen-free (SPF) eggs through the yolk sac route of the ECEs [19]. Chicken egg harvesting was performed according to OIE [20]. Titration of infectivity and EID_50_ for *C. psittaci* strain in SPF eggs were performed [21] and calculated according to Reed and Muench [22].

### Preparation of inactivated *C. psittaci* vaccine into Vero cell [23]

Vero cells were grown in a tissue culture flask containing the growth medium Eagle’s Minimal Essential Medium (MEM) with 10% heat-inactivated fetal bovine serum (FBS) (Hycolon Laboratories, Inc., USA). The flasks were incubated at 37°C and examined daily by naked eye with a tissue culture inverted microscope at 200× and 500×. The growth medium was replaced with a fresh medium when the pH dropped. When the confluent monolayer was developed, the Vero cells were dispersed with 0.25% trypsin in a calcium-magnesium-free phosphate buffer saline (PBS) 3 times at 37°C until the cells were disaggregated. Then, 1 mL FBS was added to each flask to stop the action of the trypsin. The Vero cells were then distributed into a 24-well tissue culture plate, where 5 mL of growth medium required for growth of Vero cells was added with 10% FBS to each well to stimulate growth of the Vero cells. Next, the plates were incubated at 37°C until a confluent sheet in each well was formed. The tissue culture plates were examined daily under the inverted microscope, and the media were replaced with a fresh one when the pH became acidic. The cell culture was used mainly for two purposes. The first purpose was to purify and propagate the chlamydial cells. The second purpose was to inoculate the infected cells into mice, as they must be propagated into mammalian cells such as mice cells to avoid any reactions with the ECE material.

**Figure-1:**
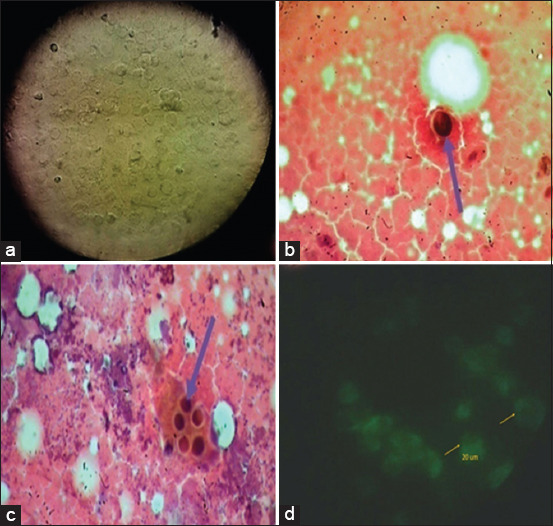
(a) *Chlamydia psittaci* Egyptian strains in Vero cell showing cytopathic effect that appears in rounding of affected Vero cells and the appearance of pyknotic nuclei. (b and c) Inclusion bodies of *C. psittaci* were detected in internal organs of the control positive group (Group E) stained with Gimenez stain. (d) *Chlamydia* direct immunofluorescence for yolk sac smears examined using fluorescein isothiocyanate showing characteristic fluorescent chlamydial bodies over the whole surface of the smear (400×).

The ECE obtained *C. psittaci* were diluted in PBS at pH 7.0 and inoculated in the Vero cells at the concentration of 10^−4^ EID_50_ per egg. The inoculated Vero cells were incubated for 5-10 days in 37°C, and then, the inoculated cells were examined using a fluorescent microscope to detect chlamydial inclusions (Figure-1a).

A seed consisting of tissue culture was harvested into sterile pooling containers. The infected mixture was centrifuged at 3000 rpm for 15 min and separated into two layers; supernatant and sediment were each inactivated with an inactivating agent (formalin 0.003%) and incubated at 37°C overnight. An immune-stimulating adjuvant, Montanide™ ISA70(SEPPIC, France), was added to the inactivated antigen to enhance its immunogenicity.

### Quality control evaluation of the prepared vaccines

Safety, sterility, purity, and stability of the prepared vaccines were all evaluated according to the US Food and Drug Administration Code of Federal Regulations [24].

### Animals and immunization schedule [25]

Ninety 2-week-old, 25 g Swiss Webster mice (Holding Company for Biological Products and Vaccines, Egypt). mice were classified into six groups of 15 mice. Group A was vaccinated with 0.2 mL of a mixture of inactivated supernatant; Group B was vaccinated with 0.2 mL of a mixture of inactivated supernatant and adjuvant; Group C was vaccinated with 0.2 mL a mixture of inactivated sediment; Group D was vaccinated with 0.2 mL of a mixture of inactivated sediment and adjuvant; Group E was the positive control mice (unvaccinated/challenged); and Group F the negative control mice received only PBS (unvaccinated/not challenged). All groups were provided with food and water. The experiment duration was 40 days from the beginning of immunization. The prepared vaccine was administered subcutaneously at the beginning of the experiment and through a booster dose after 12 days.

### *In vivo* efficacy and challenge of the prepared vaccines

A virulent *C. psittaci* strain used for challenge was kindly obtained from Dr. Eman Ragab, Faculty of Veterinary Medicine, CU and harvested in the Vero cell line. The challenge was performed using 0.2 mL of a solution containing 1×10^3^ EID_50_, of *C. psittaci* and inoculated intraperitoneally 7 days after the booster dose of the vaccine. The degree of protection was assessed according to the severity of the clinical signs and the postmortem lesions and by evaluation of the immune response [25].

### Evaluation of the immunization experiment among the vaccinated groups

#### Clinical signs

The mice in all groups were observed for the development of clinical signs and then were evaluated starting 3 days post-challenge.

### Evaluation of *C. psittaci* shedding

Nasal swabs collected from vaccinated and unvaccinated mice were put in *Chlamydia* PBS pH 7.2-7.7 medium and inoculated in ECE. Yolk sac impression smears were examined for the presence of viable *Chlamydia* by staining with Gimenez stain [26].

### Histopathological examination

Autopsy samples were taken from the liver and spleen of the vaccinated and unvaccinated mice in different groups and were fixed in 10% formol saline for 24 h. The obtained tissue was deparaffinized and stained by hematoxylin and eosin stains for histopathological examination [27].

### Direct detection of chlamydial inclusion bodies using the direct immunofluorescence technique [28]

Direct immunofluorescence enables the detection of *Chlamydia* in impression smears from the mice’s internal organs using 2 mAbs, one of which is directed against the species trachomatis and the other against the chlamydial antigen. The test was performed with *Chlamydia* Direct IF kit (BioMérieux, France), following the manufacturer’s instructions and examined under a fluorescence microscope (Olympus Microscopes, USA).

### Estimation of the serum total protein and albumin [29]

Using a commercial kit (Diamond Diagnostics, Egypt) , the total protein and albumin counts were measured in serum samples of the vaccinated and unvaccinated mice. The serum globulin level was determined by subtracting albumin from total serum protein. Measurement of globulin depended on the serum immunoglobulin concentration, which was determined depending on the concentration of total globulins and the A/G [albumin - globulin] ratio.

### Statistical analysis

Statistical analysis was conducted with an R program. One-way analysis of variance was conducted to compare the effectiveness of the used vaccine between different groups. p≤0.05 was considered to be statistically significant.

## Results

### Quality control of the prepared vaccines

The prepared vaccines were stable with no evidence of any bacterial (aerobic or anaerobic) contaminants or fungal growth after prolonged incubation (14 days) using different inoculated media. No deaths nor local or general reactions were observed in the vaccinated mice.

### Evaluation of the immunization experiment among the vaccinated mice

#### Clinical signs

**Figure-2:**
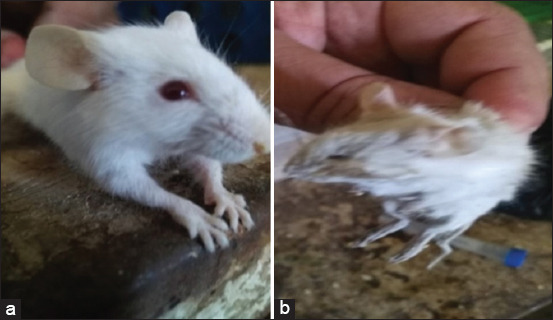
(a) Normal mice in vaccinated group showing mild signs. (b) Control positive (Group E) shows cachexia, depression, loss of weight, and eye inflammation and nasal mucoid discharges.

Clinical signs were observed in both the vaccinated and control groups at 3-9 days post-challenge, as shown in Table-1. It was clear that mild signs were observed among vaccinated groups (Groups A, B, C, and D) compared to the positive control group (Group E), Figure-2. Inclusion bodies of *C. psittaci* were detected in the internal organs of the positive control group (Group E), as shown in Figures-1b and c, while in the vaccinated Groups A, B, C, and D at 40 days post-vaccination, no inclusions could be detected.

**Table-1:** Clinical signs observed among different mice groups.

Group	Clinical signs	Number of mice showed clinical signs after challenge
Group A	Mild	3 out of 15 (20%)^A,a^
Group B	Mild	1 out of 15 (6.6%)^A,b^
Group C	Mild	6 out of 15 (40%)^a^
Group D	Mild	2 out of 15 (13.33%)^A,b^
Group E	Moderate to severe:	13 out of 15 (86.6%)^B^
	• Depression and anorexia.	
	• Cachexia and loose of appetite.	
	• Moderate-to-severe loss of body weight.	
	• Conjunctival and nasal discharges that appear to be mucoid.	
Group F	All individuals were normal, and no apparent clinical signs were observed	-

Group A=Vaccinated (supernatant+formalin)/challenged. Group B=Vaccinated (supernatant+formalin+adjuvant)/challenged. Group C=Vaccinated (sediment+formalin)/challenged. Group D=(Vaccinated sediment+formalin+adjuvant)/challenged. Group E=Unvaccinated/challenged (+^ve^ control). Group F=Unvaccinated/not challenged (−^ve^ control). Letters indicate p value significance (Capital letters A and B indicate significance between all study groups while small letters a, b, c, and d indicate significance between the four vaccinated groups)

### Evaluation of *C. psittaci* shedding

*C. psittaci* excretion was evaluated by inoculation with nasal swabs in ECEs followed by staining of the yolk sac membrane smears with Gimenez stain. *Chlamydia* direct immunofluorescence was performed for yolk sac smears, and there were characteristic fluorescent chlamydial bodies across the entire surface of the smear, as shown in Figure-1d. No shedding was detected in Groups A, B, C, or D, as no chlamydial inclusions were detected. Inclusions were detected for all examined swabs only in Group E, which all were positive.

### Histopathological examination

**Figure-3:**
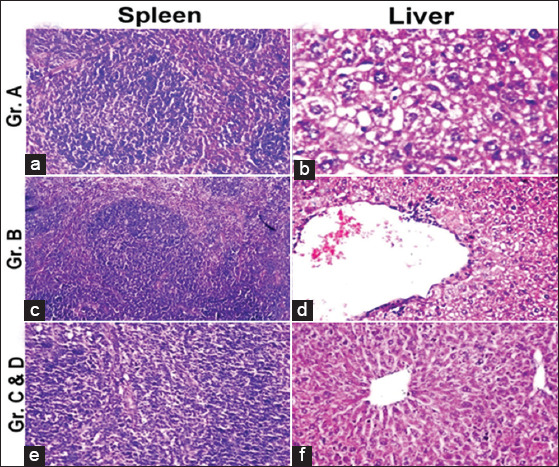
Histopathological findings among vaccinated groups showing normal histological picture of liver and spleen, summarized in Table-2.

**Figure-4:**
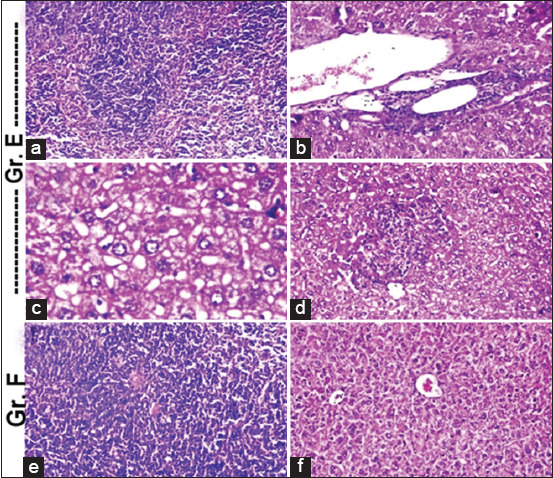
Histopathological findings among control groups showing remarkable pathological alterations were observed in liver and spleen obtained from control positive group, summarized in Table-2.

The histopathological finding from day 40 after mice euthanization is summarized in Table-2, Figures-3 and 4. The organs that were most affected macroscopically and microscopically as detected using Giemsa stain were the visceral organs (liver and spleen), which were chosen for their histopathology. All the examined tissues obtained from the negative control group showed a normal histological picture. On the other hand, remarkable pathological alterations were observed in the examined organs that were obtained from the positive control group. Otherwise, the vaccinated groups showed an observable normal picture for all of the examined organs.

**Table-2:** Histopathological findings of the vaccinated and non-vaccinated mice’s internal organs.

Groups	Liver	Spleen
Group A	No necrosis and no portal inflammatory change but there is some little fatty degeneration of liver, Figure-3b	Normal histological structure, Figure-3a
Group B	No necrosis but there are some vacuolar degenerative changes of liver, Figure-3d	Normal histological structure, Figure-3c
Group C	No portal inflammatory change or no necrosis with normal histological structure of liver, Figure-3f	Normal histological structure. With no depletion in lymphoid cells in white pulps of spleen, Figure-3e
Group D	Normal histological structure No portal inflammatory changes No necrosis (Figure-3f)	Normal histological structure. With no depletion in lymphoid cells in white pulps of spleen, Figure-3e
Group E	The portal area showed dilatation in the portal vein as well as inflammatory cells infiltration surrounding the bile ducts, as shown in Figure-4b Focal necrosis was detected in the hepatic parenchyma, as shown in Figure-4c and d, associated with vacuolar degeneration and fatty change in the hepatocytes	Lymphoid depletion in the white pulps; Figure-4a
Group F	No histopathological alteration and the normal histological structure of the central vein and surrounding hepatocytes in the parenchyma, Figure-4f	No histopathological alteration and the normal histological structure of lymphoid cells in the white pulps and red pulp; Figure-4e

Group A=Vaccinated (supernatant+formalin)/challenged. Group B=Vaccinated (supernatant+formalin+adjuvant)/challenged. Group C=Vaccinated (sediment+formalin)/challenged. Group D=(Vaccinated sediment+formalin+adjuvant)/challenged. Group E=Unvaccinated/challenged (+^ve^ control). Group F=Unvaccinated/not challenged (−^ve^ control)

### Estimation of the serum total protein and albumin

Twelve samples from each group were taken for the estimation of the total protein, albumin, and subsequently globulin to evaluate the status of infection or health conditions. The obtained values are shown in Table-3. In Groups B and C, the globulin values were elevated by from day 19 to day 40 in comparison to that of the control group (Group F) with a decreasing A/G ratio. Higher values of albumin and globulin were reached in day 33 post-inoculation.

**Table-3:** Albumin and globulin values among the vaccinated and unvaccinated mice.

Post-inoculation days (P/I)	Vaccinated groups																Control groups							
	
	Group A				Group B				Group C				Group D				Group E				Group F			
					
	TP	A	G	A/G	TP	A	G	A/G	TP	A	G	A/G	TP	A	G	A/G	TP	A	G	A/G	TP	A	G	A/G
Begin of vaccination																								
Day 0																								
Day 19	13.6	3.1	10.5	0.3	22.7	4.3	18.4	0.2	18.8	3.9	14.9	0.3	13.5	2.9	10.6	0.3	2.9	1.5	1.4	1.1	2.2	1.3	0.9	1.4
Begin of challenge																								
Day 26	17.9	3.7	14.2	0.3	29.3	4.4	24.9	0.2	30.2	3.2	26.9	0.1	16.4	3.0	13.4	0.2	4.6	3.0	1.6	1.9	2.1	1.4	0.7	2.0
Day 33	27.3	4.7	22.6	0.2	33.7	5.0	28.7	0.2	32.7	4.6	28.2	0.2	19.2	3.1	16.0	0.2	3.8	3.0	0.9	3.5	2.5	1.6	0.9	1.9
Day 40	17.3	3.6	13.6	0.3	31.6	4.4	27.2	0.2	30.5	3.4	27.1	0.1	19.3	2.5	16.7	0.1	3.7	1.5	2.2	0.7	1.9	1.5	0.4	3.8

In Groups A and D, the albumin and globulin values increased but were less than in Groups B and C in comparison with those of the control group, which decreased in the A/G ratio. High values of albumin and globulin were recorded in day 33 post-inoculation but were low on day 40. In Group E, albumin values decreased with the elevation of globulin values in comparison to those of the control group with an increasing A/G ratio.

### Post-challenge protection percent verification

All mice were kept under observation and the clinical signs were recorded. No local reaction was found in all vaccinated mice (S/C injection). There were significant differences between the clinical signs of vaccinated and those of the unvaccinated groups. The number of mice showing clinical signs after challenge was noticed to be higher for the unvaccinated mice (Group F, 86%) compared to that among vaccinated mice (Groups A, B, C, and D were 20%, 6.7%, 40%, and 13.3%, respectively) (Table-1).

## Discussion

C. psittaci is the pathogen of psittacosis, and it has emerged as a significant public health threat [30]. It is an intracellular bacterial species that may cause several infectious systemic and often lethal diseases in a wide range of poultry and wild birds (endemic avian chlamydiosis) [31], as well as epizootic outbreaks in mammals [32].

The microorganism has prevalence in public health, as it can transmit to humans by diseased or even asymptomatic birds causing sporadic but sometimes devastating disease, such as “psittacosis disease,” with transmission occurring through inhalation, ingestion, or skin contact to eyes [33].

*C. psittaci* infection constitutes many issues that impact its severity. It has a broad range of hosts and causes significant economic losses in the poultry industry. Birds may be an asymptomatic carrier capable of transmitting the pathogen zoonotically. It is difficult to diagnosis and poses a public health hazard, so controlling chlamydial infections are critical to bird and human safety [34].

Despite chlamydial infections being susceptible to antibiotic treatment, vaccination still appears to be the most desirable approach for disease control, because it overcomes the inability to give antibiotics to all apparent healthy carriers who shed the organism in secretions and fecal matter, which leads to continuous infection in a latent state, which under stress conditions can result in disease recurrence. Until now, vaccines have been available for only two species, *C. abortus* and *C. felis*, which infect ovine and feline species, respectively [35].

The development of an effective vaccine against *C. psittaci* will protect susceptible poultry from infection and production performance losses, reduce the zoonotic risk, and minimize the emergence of antibiotic-resistant *C. psittaci* strains [36].

The challenge of developing a vaccine against *Chlamydia* species that is a cheap killed or inactivated organism that effectively provides long-term protection and is safer to use than the live-attenuated vaccine is to avoid a change of the attenuated strain into a virulent one [14]. Yu *et al*. [37] conducted mass spectrometry experiments to detect proteins in the chlamydial outer membrane complex from the elementary body of *C. muridarum*. They suggested that conformational intact proteins will be necessary for the efficacy of a recombinant outer membrane protein vaccine. The signal peptide vaccine, which is based on Pgp3-dominant epitopes, plays a significant role with good immunogenicity and protective efficacy against *C. psittaci* lung infection in BALB/c mice [30].

The present study tested the efficacy of the inactivated whole bacterin vaccine against *C. psittaci* prepared in tissue cell lines and experimentally inoculated in mice. Three Egyptian sequenced strains were selected, grown in ECE, inoculated in Vero cell lines, and inactivated with formalin. Montanide (M-ISA-70) was added as the effective adjuvant to slow the release and give long immunity, especially in pregnant mothers to provide maternal immunity. The quality control of our prepared killed vaccine was studied, and it was pure, sterile, and safe according to the US Food and Drug Administration Code of Federal Regulation [24].

The efficacy of the prepared vaccines was experimentally conducted in 90 mice that were classified into six groups of 15 mice. The first four groups (A-D) were vaccinated, while the fifth and sixth groups were left unvaccinated as the positive control and the negative control, respectively. There was a protection pattern in the form of a variation in the degree of clinical signs between vaccinated, challenged, and control groups. Medium-to-severe clinical signs were observed in 13 out of 15 mice (86.6%) in the positive control Group E. The shedding test also revealed positive Giemsa samples from Group E. Furthermore, the histopathological findings of the vaccinated groups demonstrated no or minimal alterations in contrast to the severe clinical signs that were recorded for the positive control group.

The presence of *C. psittaci* in the tissues of the immunized mice was evaluated using direct immunofluorescence. *Chlamydia*l inclusions were found in all groups with varied percentages, except in Group F (−^ve^ control) and Group B, which were vaccinated with supernatant+formalin+adjuvant. Indirect fluorescein-conjugated anti-mouse antiserum in combination with the mAb staining procedures that utilize mAbs directed against chlamydial surface antigens was more sensitive and produced more striking results in comparison to that of histochemical staining [38,39].

An estimate of the globulin was used to evaluate the status of immunity against infection or the degree of health condition by estimating total protein and albumin. It is clear that the globulin values were elevated from day 19 to day 40 (the maximum value was day 33) in vaccinated mice Groups B and C, and to a lesser extent in Groups A and D in comparison to the negative control group (Group F) as the A/G ratio decreased. In the positive control group (Group E), the elevated globulin values with low plasma albumin values suggested liver injury [40].

The complete evaluation of the vaccinated groups in comparison to both the negative and positive control groups indicated that the best protection was achieved with the administration of formalized supernatant with adjuvant (Group B) or formalized sediment with adjuvant (Group D), while the less effective groups were those that had been administered the non-adjuvant vaccines (Groups A and C). These results demonstrate the importance of adding adjuvant to the prepared vaccine to improve its efficacy and immune response.

Waldhalm *et al*. [41] compared the efficacy of *C. psittaci* bacterin prepared in the mouse L-cell line with a similarly prepared bacterin grown in chicken embryo (CE). It was declared that both bacterins significantly reduced the incidence of abortion and weakness in lambs compared to that of the non-vaccinated control ewes. Notably, the L-cell bacterin elicited a greater antibody response than did the CE bacterin. Rank *et al*. [42] immunized female guinea pigs with UV light-inactivated *C. psittaci* by different routes: Intravenous, subcutaneous, oral, and ocular routes. Then, all the animals were challenged vaginally with viable chlamydia. The genital infection was intensely reduced for all groups of animals except for the unimmunized controls and those animals immunized orally. A commercial yolk sac-derived adjuvant formaldehyde-inactivated C. abortus strain was found to be effective against ovine enzootic abortion in sheep for as long as 20 years until the disease appeared again in the vaccinated ovine flocks [14].

The main drawback of using a killed or inactivated vaccine in case of immunization against *C. psittaci* is that the immunity to the intracellular pathogen has been thought to be dependent on the cellular arm of the immune system. However, tissue examination studies often revealed that the intracellular microorganisms could be found in the extracellular space, where they are vulnerable to antibody action. Furthermore, Fc receptor cross-linking can have profound effects on the intracellular milieu through signal transduction [43,44].

## Conclusion

The significant economic impacts and financial losses resulting from avian chlamydiosis, especially those in poultry industries, along with the zoonotic transmission risk highlight the need for proper disease control measures. The killed vaccines used in this trial successfully overcame the disadvantages of using live-attenuated, recombinant, and DNA vaccine techniques. The immunization trial revealed that an inactivated or killed vaccine effectively reduced chlamydial shedding from poultry with an adequate level of protective antibody titer, which creates an effective barrier for protection. The development of an inactive or killed vaccine that is capable of protecting against chlamydial infection presents a special challenge, but it would be the most effective long-term option for the control of chlamydial diseases.

## Authors’ Contributions

JE, MDE, and MAE have designed the plan of work, supervised the experiment, and revised the manuscript writing. ER, ESG, EAK, RHH, and AAK shared in all vaccine preparation and evaluation steps. All authors read and approved the final manuscript.
